# A multi-level multi-scale approach to study essential genes in *Mycobacterium tuberculosis*

**DOI:** 10.1186/1752-0509-7-132

**Published:** 2013-12-05

**Authors:** Soma Ghosh, Priyanka Baloni, Sumanta Mukherjee, Praveen Anand, Nagasuma Chandra

**Affiliations:** 1 Department of Biochemistry, Indian Institute of Science, Bangalore, India

**Keywords:** Essential genes, Multi-scale analysis, *Mycobacterium tuberculosis*, Protein structure

## Abstract

**Background:**

The set of indispensable genes that are required by an organism to grow and sustain life are termed as essential genes. There is a strong interest in identification of the set of essential genes, particularly in pathogens, not only for a better understanding of the pathogen biology, but also for identifying drug targets and the minimal gene set for the organism. Essentiality is inherently a systems property and requires consideration of the system as a whole for their identification. The available experimental approaches capture some aspects but each method comes with its own limitations. Moreover, they do not explain the basis for essentiality in most cases. A powerful prediction method to recognize this gene pool including rationalization of the known essential genes in a given organism would be very useful. Here we describe a multi-level multi-scale approach to identify the essential gene pool in a deadly pathogen, *Mycobacterium tuberculosis*.

**Results:**

The multi-level workflow analyses the bacterial cell by studying (a) genome-wide gene expression profiles to identify the set of genes which show consistent and significant levels of expression in multiple samples of the same condition, (b) indispensability for growth by using gene expression integrated flux balance analysis of a genome-scale metabolic model, (c) importance for maintaining the integrity and flow in a protein-protein interaction network and (d) evolutionary conservation in a set of genomes of the same ecological niche. In the gene pool identified, the functional basis for essentiality has been addressed by studying residue level conservation and the sub-structure at the ligand binding pockets, from which essential amino acid residues in that pocket have also been identified. 283 genes were identified as essential genes with high-confidence. An agreement of about 73.5% is observed with that obtained from the experimental transposon mutagenesis technique. A large proportion of the identified genes belong to the class of intermediary metabolism and respiration.

**Conclusions:**

The multi-scale, multi-level approach described can be generally applied to other pathogens as well. The essential gene pool identified form a basis for designing experiments to probe their finer functional roles and also serve as a ready shortlist for identifying drug targets.

## Background

An *essential gene* is defined as a gene necessary for the growth and maintenance of an organism [1]. Identifying a set of *essential genes* for a given condition, is of significant importance for a number of reasons: (a) for understanding pathogen biology: identification will help in prioritizing the set of genes for functional studies; (b) from an evolutionary perspective: since a distinct correlation is suggested between essentiality and extent of conservation in a given family or class of organisms [2]; (c) in drug discovery: essential genes in a pathogenic species form a ready shortlist of possible drug targets [3]. Essentiality to the pathogen is in fact one of the key criteria for defining a drug target [4]; (d) essentiality also serves as a useful parameter in biomarker identification, since by definition, an essential gene is necessarily present in the pathogen [5]; (e) from a synthetic biology perspective, an essential gene set should overlap extensively with the minimal gene set required for survival of the organism and hence an identification of the essential set also forms a starting point for future work towards synthetic reconstruction of the organism [6]. As whole genome sequences of a large number of species are being deciphered and *omics* data covering various aspects are accumulated, a number of both experimental and computational methods are being explored to identify essential genes [7-11]. There are reports based on functional genomics studies of several organisms, which indicate that only 15 - 20% of the genes are essential to the organism under a given condition [12].

Approaches used for identification of essential gene set include classical forward genetic screens [13], genome-wide RNA interference screens [14] and targeted gene knockouts [15]. Typically, a given gene is deleted or inactivated by one of the listed techniques and if the resulting mutant strain leads to ‘loss of function’ , in terms of the loss of viability of the organism, the given gene is said to be essential. Whole genome transposon mutagenesis, which involves exploitation of transposon or mobile DNA elements, as mutagens, so as to inactivate the gene, has been applied to a number of organisms [16-19]. The basic premise in these is that, the inability of the bacterium to survive due to the disruption of gene expression by the inserted transposon indicates essentiality of the disrupted gene. Databases such as DEG [20], and OGEE [21] have also been developed, that combine information about essential genes in a number of organisms based on extensive literature survey. While, each of these methods has tremendous advantages, they come with their own limitations as well and hence cannot be expected to identify essential genes comprehensively. Even when successful, these techniques do not provide any mechanistic insights about why a particular gene is essential.

Computational methods to probe essentiality have mainly involved identification of orthologs in related organisms and assessing phyletic retention. Sequence features such as GC-content, codon usage, and localization signals have also been used for inferring essentiality, although these have been found to be less accurate [1,22]. Rio and coworkers have reported a method for identification of essential genes from large interaction networks and report significant prediction accuracy by using a combination of network parameters and centrality measures [8]. Plaimas and coworkers [11] apply a machine learning approach to identify essential genes in bacterial metabolic networks, wherein features characterizing network topology, sequence information and co-expression profiling were utilized for training, which was further applied to identify drug targets in *Salmonella typhimurium*. Constraint-based metabolic modeling approaches such as flux balance analysis have been used to analyse genome-scale metabolic networks in several organisms such as *Staphylococcus aureus*, *Helicobacter pylori*, *Mycoplasma genitalium* and *Pseudomonas aeruginosa*. Simulation of virtual knockouts of genes in these networks is helpful in inferring essentiality [23-26]. Very recently *Karr et al.*[27] have built a whole cell computational model of the life cycle of *Mycoplasma genitalium*, wherein the cell was divided into different modules based on the functional capacity of each module. Each module was then independently modeled using mathematical tools that best suits the model: FBA for metabolism, Poisson processes for RNA and protein degradation. The built model thus could accurately reproduce experimental data and provide insight into many biological processes. Such studies highlight the importance of integrating different mathematical or computational aspects for different biological processes. Different computational methods capture different aspects that define why a gene is essential and hence, no method individually is sufficient to provide a powerful predictive tool, warranting exploration of newer approaches for studying essentiality.

Essentiality is inherently a systems property [28]. A gene known to have an important function may not be essential in the whole system due to genetic redundancy or functional pleiotropy [29]. The loss of some genes can be compensated by alternate pathways to reach the same biochemical goal. In other cases the function of the gene may not be essential for growth or survival, but may have some specialized function such as imparting virulence [30]. A systems approach, therefore, becomes necessary to address these issues.

There are nearly two million deaths every year, translating to one death every 15-18 seconds, due to tuberculosis [31]. Tuberculosis (TB) has unfortunately retained the status, for number of decades, as being the leading killer among all infectious diseases. The strong synergy of the causative organism *Mycobacterium tuberculosis* (*M.tb*) with the deadly virus HIV has made the problem more acute. Although a handful of drugs and vaccines are available for the treatment of this disease, the problem has remained acute due to difficulties in diagnosis, long periods of treatment, inability to tackle latent forms of the bacteria and more importantly the emergence of drug resistant varieties of *M.tb* such as MDR, XDR and TDR strains [32]. Further research to understand the biology of the pathogen in a more wholistic approach and to apply the knowledge for the identification of newer and more efficient drug targeting strategies is thus urgently required. The whole genome sequencing of *M.tb*[30], about 15 years ago has triggered intense functional genomics studies on *M.tb*, leading to the accumulation of several types of *omics* data. Several computational studies are also available [33,34], adding to the resource base, making it feasible to address complex issues such as gene essentiality for this pathogen from an integrated perspective. Methods to integrate various *omics* data into networks are also beginning to be described in literature [35].

Here we seek to study gene essentiality at multiple levels and at multiple scales of spatial resolution, to identify with high confidence, a set of essential genes in *M.tb*. The study uses a range of models at each level, including genome-scale interactome, metabolic network, individual pathways and biochemical reactions and finally individual amino acid residues in the proteins. Computational approaches at systems, structural and sequence levels have thus been employed.

## Results

**Figure 1:**
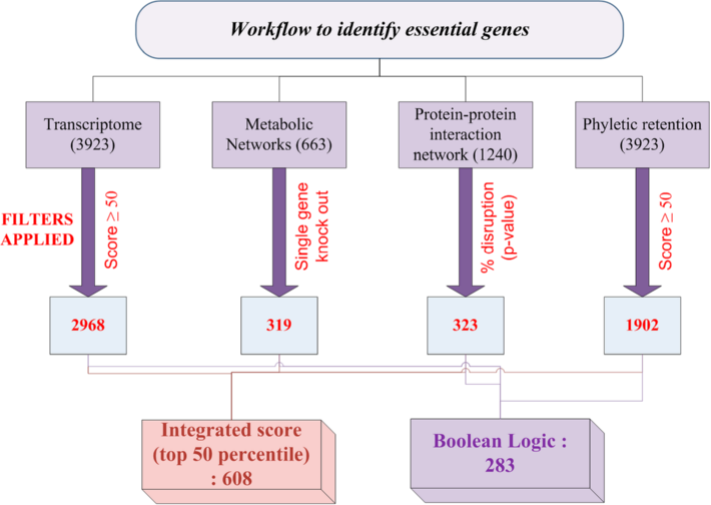
**Workflow of the method used in this study.** The figure represents the pipeline that has been followed in this study to identify essential genes. Four different methods have been applied independently to filter probable candidates for essentiality. The results thus obtained have been integrated using Boolean logic to obtain a final list of essential genes. In addition, integrated scores have also been assigned to each gene. The numbers in the bracket represents the initial number of genes analysed using the different methods, which are further filtered.

A new multi-level approach has been used to study gene essentiality. In brief, experimentally derived gene expression data from literature has been analysed to identify genes exhibiting consistent expression patterns in about 39 different sets of the same condition. Next, flux balance analysis (FBA) of a genome-scale metabolic model integrated with gene expression values, has been carried out to identify essential genes through systematic, *in silico* gene knockouts (KOs). Further, a directed, weighted genome-scale protein-protein interactome has been constructed and analysed to identify key points controlling the topology of the network. A large-scale sequence analysis has been carried out to test for those genes that have high phyletic retention in genomes of the same genus. The workflow followed in this study is illustrated in Figure 1 and describes the different methods and filters that have been employed to derive essentiality. The set of essential genes so identified are analysed, using multiple sequence alignment, of the corresponding protein sequences, to obtain conserved amino acid residues which are then mapped on the functional sites in the protein to determine the basis of essentiality. Put together, this study helps in addressing essentiality at the levels of modules in the network, pathways and at the level of individual proteins. To our knowledge, such an integrated approach has not been extensively explored earlier.

### Microarray data analysis

Microarray analysis provides a global picture of the expression profiles of all the genes in an organism under a given condition. It is reasonable to assume that an essential gene ought to be expressed in the cell in sufficient quantities. Although, expression by itself does not dictate function in a bacterial cell, consistent expression patterns of individual genes can be considered to provide a rough indication of the functional capacity of that gene in the cell. In this study, genes that exhibit consistent and high expression patterns in many samples of the same wild type (WT) growth condition are grouped into a shortlist of probable candidates for essentiality. This is based on the premise that a cell expends its limited energy and resources towards protein synthesis only if the protein has certain functional significance for the survival of the cell [36].

Microarray based gene expression dataset considered for the present study consists of a total of 39 samples of the same WT condition, with 3923 genes in each sample [37]. A scoring scheme was devised for all the genes in the genome that forms a continuous scaling measure (Equation 4). The score captures consistent expression in the 39 different microarrays used for the study and for a given expression level, those which show consistent expression, will have the highest scores. It is important to note here, that while high expression is not a necessary condition for the gene to be termed essential, consistent expression across biological samples of the same kind is required for a gene to be considered for essentiality. Jeong *et al.*[38] have earlier shown the importance of consistency in gene expression data for predicting gene essentiality in yeast, *Saccharomyces cerevisiae*.

In this work, genes which appear up to the 50^th^ percentile in the ranked list (based on Equation 4) were selected for further analysis. At this stage, since the intention is to obtain a shortlist for further analysis in the pipeline, a lenient cut-off of 50^th^ percentile was chosen so as to avoid any false negatives. As many as 2968 genes (listed in Additional file 1: Table S1) out of 3923 genes were identified by this criteria indicating that a large number of genes showed consistent and significant levels of expression. Although it is theoretically possible to miss some genes that may be consistently expressed at very low levels, the rather broad cut-off used at this stage implies that whatever maybe missed will only be a small fraction, since of the 955 genes eliminated, most (97.5%; 932 out of 955) showed generally low expression and large variations (SD > 0.5).

These 2968 genes were further classified into different Tuberculist [39] functional classes. As can be seen from Additional file 2: Figure S1, genes involved in metabolism and respiration form a major portion of the pie-chart (24%). *M.tb* is known to have developed sophisticated mechanisms for cell-wall synthesis which is also reflected in the figure, with 20% of the genes falling in this category. 24% of this set is formed of conserved hypotheticals, highlighting the knowledge gaps that exist for *M.tb*. About 10% of the genome from this list is dedicated for PE and PPE families of proteins, which are unique to mycobacteria [30]. The functions of some of these are now understood and it is believed that they are important for virulence and antigenic diversity of the pathogen [40]. Variable patterns of gene expression for these genes during infection have been discussed earlier [41,42]. These set of proteins are involved in the modulation of antigen processing and presentation and thus very successful in evading immune responses. These set of genes have been reported as immune-dominant antigens and are ideal drug targets. About 4% of the shortlisted genes belonged to this functional category.

### Flux balance analysis

Metabolism is a fundamental requirement in an organism to sustain life. Flux balance analysis (FBA) captures the metabolic state of a system at steady state condition. This is achieved by reconstructing a genome-scale metabolic network [43] and computing the relative fluxes of individual biochemical reactions within the constraints of mass balance and defined stoichiometric coefficients of the network, so as to obtain maximal growth. The method enables systematic perturbations through simulation of KOs of individual genes in the model. An essential gene and hence the protein can easily be identified through simulations that lead to zero or reduced growth [44].

Genome scale metabolic reconstruction for *M.tb* H37Rv used here consists of 661 genes and 939 reactions [45] and is referred to as *i*NJ661. Gene expression values were incorporated into this model to guide the bounds of the individual reaction fluxes. The method of incorporating gene expression values is based on E-Flux reported in the literature [46]. Bounds for each reaction were assigned based on the Gene-Protein-Reaction relationships (GPRs) provided with the model. Integrating expression data into FBA makes the network much more biologically realistic, since dynamics of a metabolic network would also depend on the protein concentrations available for the reaction to occur. Fluxes are calculated through each reaction so as to maximise the biomass function. The objective value obtained for the original model was 0.0398 hr^-1^, while for the modified model it was 0.0039 hr^-1^. Around 46 - 47% non-zero flux reactions were obtained in both the cases. Single gene deletions were performed by systematically deleting each gene in the model and calculating the optimal fluxes using the same biomass function. The ratio of the objective value of the KO and WT (grRatio) was calculated and plotted for the original and the modified model. The value of grRatio determines the importance of a particular gene for the growth of the organism and hence can be used to predict essentiality.

**Figure 2:**
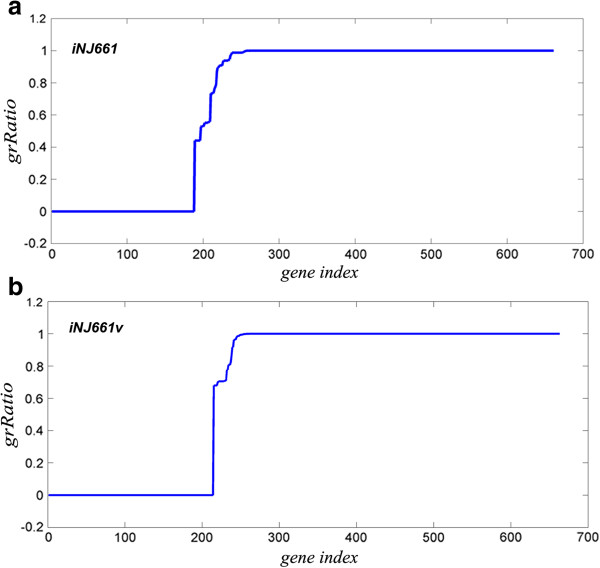
**Flux Balance Analysis for the metabolic model.** Single gene deletion profile of the gene expression integrated FBA model. X-axis represents the gene index and the y-axis is the ratio of the objective value in KO to the WT (grRatio). The gene index is sorted based on the ratios obtained for **a) ***i*NJ661 model and **b) ***i*NJ661v model.

In the original model that did not consider expression data, KOs of 230 genes affected the optimal objective function, out of which 188 were lethal and the remaining showed transitional effect. By adding microarray data, the result did not change much, except for an increase in the number of genes affecting the objective function. A total of 260 genes were affected, with 188 lethal KOs and the rest showing reduced growth as compared to the wild type (Figure 2a).

*i*NJ661 and GSMN-TB [47] are two metabolic networks reported almost simultaneously, both sharing a high amount of similarity. Besides these, newer models have been developed recently for the same organism with more number of reactions and genes. One such model is *i*NJ661v [48] which contains 663 genes, 838 metabolites, and 1,049 reactions that incorporates reactions from both *i*NJ661 and GSMN-TB, and shown to be more sensitive towards predicting essentiality. The objective value obtained for the model was 0.07 hr^-1^, with 49.85% of the reactions showing non-zero fluxes. Therefore, in addition to *i*NJ661 model, *i*NJ661v model was also considered for analysis. The method for predicting gene essentiality was similar to the *i*NJ661 model, and this lead to the addition of 59 more genes to the already identified 260 genes. The grRatio for KO simulations for this model is shown in Figure 2b and a comparison of the impact of gene deletions in the two models is shown in Additional file 3: Figure S2.

The genes obtained were further classified into functional groups using the scheme provided in Jamshidi *et al.*[45]. As seen in Additional file 4: Figure S3(a), genes from membrane metabolism (12%) and fatty acid metabolism (9%) were significant, reiterating the importance of lipid metabolism and cell wall synthesis in *M.tb*. Purine metabolism (10%) and redox metabolism (7%) also contribute significantly to this set of genes.

Apart from the single gene deletions, which characterised genes required for the survival of the organism, double gene deletions were also performed to look for pairs of genes which when knocked out simultaneously are lethal or unfavourable to the organism. 110 such gene pairs (Additional file 5: Table S2) were obtained and could serve as potential drug targets. Additional file 4: Figure S3(b) gives the pathway classification of the genes identified from double KO analysis. In this case, genes belonging to fatty acid metabolism (19%) account for higher contribution, followed by citric acid cycle pathway (14%) and purine metabolism (8%) while glutamate metabolism and glycine, serine, threonine metabolism account for 7% of the total list. About 6% of genes are involved in redox metabolism and 5% of genes contributed to pyruvate metabolism and transport respectively.

From this analysis, 319 genes (hypergeometric p-value < 0.0001) which showed lethal or unfavourable growth on performing single gene deletions for the gene expression integrated models were selected as candidate essential genes (Additional file 1: Table S1). The hypergeometric test was performed to establish the significance of the subset of genes identified as essential from all the genes analysed using FBA. The genes identified as essential through this approach were later categorised into different functional classes based on the schema provided by Palsson and co-workers [45].

### Network analysis

Protein–protein interaction networks are known to capture global as well as the local behaviour of a system, despite having the limitation of being static in nature. This can be resolved to some extent by integrating gene expression data into the network and then deriving various insights through calculation of network properties [38]. Shortest paths between all pairs of nodes in a network serve as useful pointers to understand the overall topology of the network and the extent of interaction between the nodes [49]. Adding weights based on expression level of a node leads to construction of response networks and hence identification of biologically significant paths [50].

An extensively curated, weighted and directed protein–protein interaction network was generated using standards described in the methods section. It consisted of a total of 1240 nodes and 7844 edges (Additional file 6: Table S3). This was a high confidence network and an interaction was included only if sufficient evidence was available for that interaction in literature and databases. To make the network more biologically significant, microarray data was integrated into the network as node weights. Each edge was assigned a weight that was a function of the betweenness centrality of that edge and the node weights connecting that edge (Equation 5). Shortest paths were exploited to study the importance of each protein by performing systematic KOs in the network. Importance of a gene or protein is then simply a measure of the number of shortest paths disrupted upon knockout of that node in the network. Number of disrupted paths also indicates whether a given protein causes a global disruption to the network or whether it brings about a local effect.

**Figure 3:**
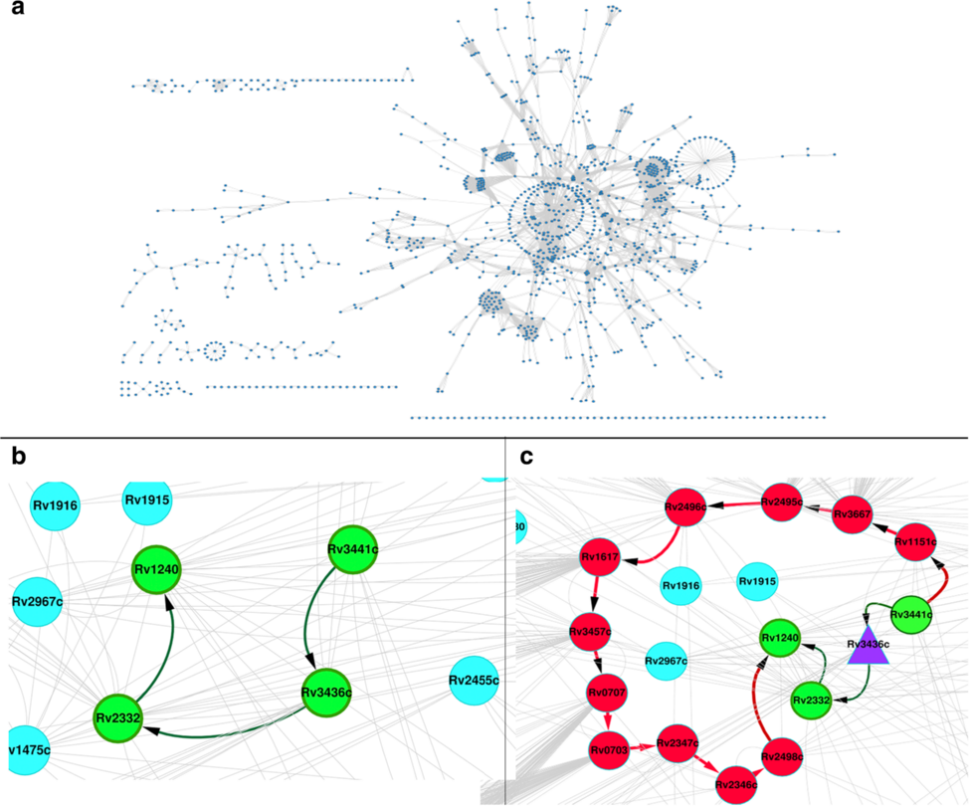
**Network topology of protein-protein interactome and an example gene KO analysis. a)** Shows the overall topology of the protein-protein interaction network used in this study. The network consists of 1240 nodes and 7844 edges. Essential genes were identified by systematic KO of each node in the network and by calculating the number of paths perturbed; **b)** illustrates the path between Rv3441c (mrsA) and Rv1240 (mdh) in the WT network. Nodes involved in the path are coloured green. **c)** Represents an alternate (but unlikely to be feasible) path between Rv3441c (mrsA) and Rv1240 (mdh), when Rv3436c (glmS) is knocked out. The purple triangle shows the node which is knocked out. Green nodes represent the original path in the WT network and red nodes are the reprogrammed alternate path that the system takes upon KO.

For each KO performed, the number of completely broken paths as well as the number of paths perturbed as a result of increased path cost was calculated. A path cost is taken as the summation of the edge weights defining that path. Perturbed paths are indicative of the alternate paths that emerge as a result of that KO and invariably have a higher path cost as compared to the WT. An example of this is illustrated in Figure 3. Figure 3b demonstrates an example of the shortest path between Rv3441c (mrsA) and Rv1240 (mdh) in a WT network. This path includes genes Rv3436c (glmS) and Rv2332 (mez) and has path cost equal to 6.36 × 10^-5^. All the four genes are involved in cellular metabolism according to Tuberculist [39] and KEGG [51] annotations. Detailed list of annotations are provided in Additional file 7: Table S4. Upon KO of Rv3436c (glmS), the simulation indicates that the next best theoretically possible path (Figure 3c), is much longer than the original path and also has a higher path cost, 2.6 × 10^-4^ which is about 10 times higher than the WT path cost. Such a significant increase in the path cost indicates that this alternate path may be biologically infeasible and thus the node Rv3436c would be essential to maintain original function. The perturbed path comprise 13 nodes (Rv3441c (mrsA) → Rv1151c → Rv3667 (acs) → Rv2495c (bkdC) → Rv2496c (bkdB) → Rv1617 (pykA) → Rv3457c (rpoA) → Rv0707 (rpsC) → Rv0703 (rplW) → Rv2347c (esxP) → Rv2346c (esxO) → Rv2498c (citE) → Rv1240 (mdh)), including the source and destination.

**Figure 4:**
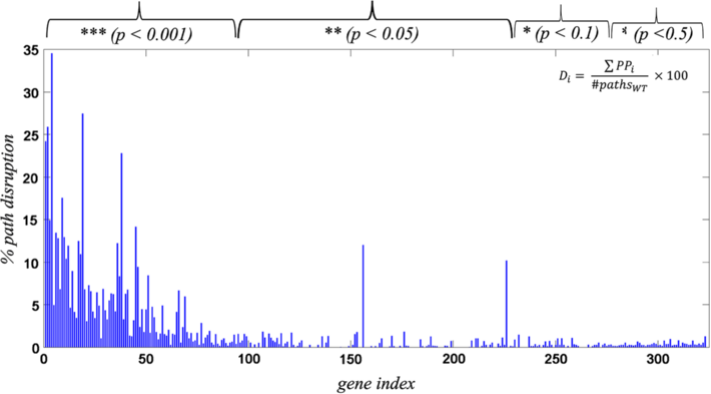
**Broken Path Analysis of the protein- protein interaction network.** Effect of a KO in the network was determined by calculating the percentage disruption in the network based on the number of paths perturbed. The plot represents the% disruption of each gene knockout on the y- axis, while x - axis represents the gene index sorted based on p values. Also shown is the significance star for the genes based on the p values calculated using random networks.

Broken path analysis was performed to study the effect of gene deletion on the network. Systematic knockout was performed to obtain percentage disruption by each gene. A total of 323 genes (Additional file 1: Table S1) when knocked out showed broken paths in the network. To obtain the significance of these KOs p-values were calculated using random networks. Generation of random network is described in the methods section. It was observed that for a majority of the 323 genes, the p-values were < 0.05, while few were in the range of 0.1 to 0.5. The percentage disruption of each of these 323 genes, as calculated by equation 6 in the methods section, is plotted in Figure 4, and the gene indexes are sorted by p-value on the x-axis. It is also noted that the number of paths perturbed for each KO may not necessarily correlate linearly with an increase in total path cost for that KO. This is quite understandable since the impact of perturbation of different paths is not uniform. To capture the relative importance of a perturbation due to a KO, we have ranked nodes based on number of paths perturbed. Some KOs are seen to cause as much as 34% disruption in the network. Such nodes are considered to be critical control points in the network. A significant portion (63.5%) of these belongs to the functional category of intermediary metabolism and respiration.

### Phylogenetic analysis

Conservation of a protein across species can also broadly imply essentiality as they have been retained through evolution. BLAST [52] searches were performed for all the *M.tb* H37Rv sequences against a genus-specific dataset of 63 species, representing a set of evolutionarily related organisms. The dataset was prepared by considering all *Mycobacterium* species, whose whole genome sequences were available, excluding all the *M.tb* strains. Thus, this dataset includes species that are pathogenic, non-pathogenic, fast and slow growers as well as those pathogenic to non-human hosts. Sequences with identity ≥ 30% with sequence coverage ≥ 70% and e-value of ≤ 0.001, with respect to the query protein were chosen for studying essentiality.

Proteins showing high phyletic retention were shortlisted. Quantitatively, a score was assigned to each protein based on Equation 7, explained in the methods section. Those with a score ≥ 50 were selected to be essential, which in turn implied that the gene is conserved in at least 50% of the species in the mycobacterial genus. 1902 sequences were thus identified and are listed in Additional file 1: Table S1.

A functional classification of this set was also performed based on Tuberculist classification scheme (Additional file 8: Figure S4) and it is observed that large number of genes belong to intermediary metabolism and respiration (29%), conserved hypotheticals (23%), cell wall and cell processes (19%) and lipid metabolism (11%).

### Deriving the final list of essential genes (EGs) and comparison with experimental datasets

**Figure 5:**
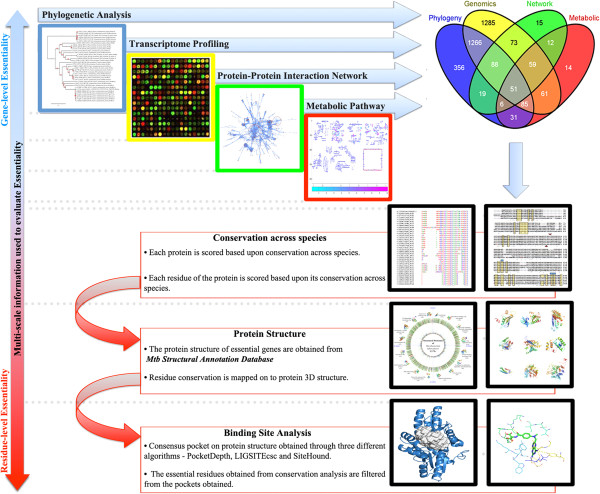
**Integration scheme used in this study.** Different levels of abstractions used in this study to obtain essential genes are shown. Different methods are highlighted with different colours as shown in the top panel, phylogenetic analysis (blue), transcriptome profiling (yellow), protein-protein interaction (green) and metabolic analysis (red). All the methods are integrated using set theory to obtain a list of high confidence essential genes as shown in the Venn diagram. The final set of essential genes is further analysed to probe into residue level conservation and binding site analysis (lower panel).

Different methods in this study capture gene essentiality with different perspectives as illustrated in Figure 5. FBA provides information based on metabolite flow, while phylogenetic analysis captures essentiality based on evolutionary conservation. Topological networks provide an overview of connectedness among various proteins while microarrays add quantitative information about the relative abundances of different genes. Integrating them together will enable us to address essentiality from all these perspectives simultaneously. Towards this, set theory was applied as follows:

(1) $$\mathrm{EG}=\left(\left(F\cap N\right)\cup \left(F\cap P\right)\cup \left(P\cap N\right)\right)\cap M$$

Where,

*EG* = essential genes,

*F* = genes identified using gene expression integrated FBA,

*N* = genes identified using network,

*P* = genes identified using phyletic retention,

*M* = genes identified using microarray,

The Boolean logic (Equation 1) selects those genes that were predicted as essential by at least two of the three methods (FBA, phyletic retention and network analysis) in addition to the necessary condition of consistent expression pattern. The rule considers expression data as an essential but not a sufficient condition to predict essentiality. If a gene is considered essential, it is important that it expresses sufficiently and consistently. A final set of 283 genes were identified using Equation 1 and is provided in Additional file 9: Table S7.

An earlier study had experimentally identified a set of 654 genes, of which 614 were considered as essential while 40 more were found to disrupt the growth of the organism using transposon site hybridization method [18]. The same group has subsequently used high density mutagenesis and deep sequencing methods to identify a set of 774 genes (p < 0.05) as essential [16]. The latter is reported to be a more sensitive method and apart from identifying those reported by the former approach, it adds many new ones. A recent study by Zhang *et al.*[19], that identified essential genes by dividing the whole-genome sequence into windows of different length and studied essentiality by performing TraSH analysis have also become available. Thus, the set of 283 genes obtained from our study is compared to these datasets, from which we observe an agreement for 182 genes. A few other datasets of experimentally identified EGs under different conditions or using different approaches are also available [17,53-60]. From these, an agreement is observed for another 26 genes. Put together, the agreement with our analysis is around 73.5%. It must be noted that the experimental approaches too have their limitations and fail to capture essentiality in some situations. Despite these limitations, it is encouraging to observe a high correlation with these datasets.

**Figure 6:**
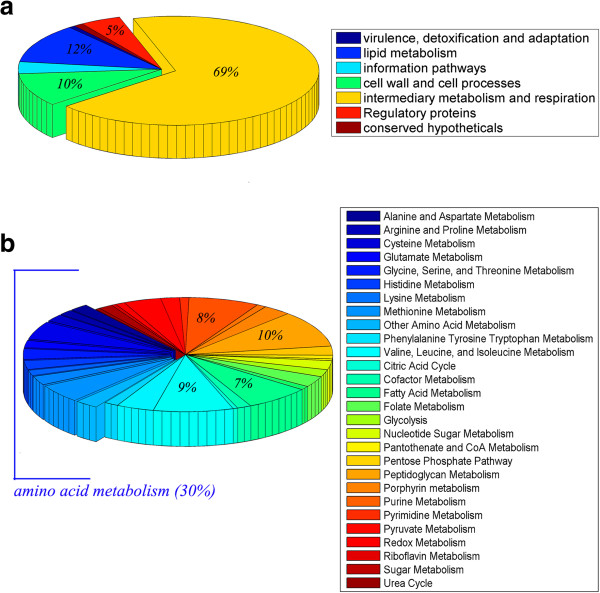
**Functional classification of essential proteins identified. a)** Shows the functional classification of the final list of essential gene based on Tuberculist. **(b)** About 69% of the genes belong to metabolism which has been further classified using the scheme given in [45].

Functional classification of the identified 283 genes was carried out using the Tuberculist scheme and is shown in Figure 6a. About 69% (24%) belonged to metabolic pathways while 12% (6.9%) and 10% (19.44%) was obtained for lipid metabolism and cell wall processes respectively, accounting for the importance of these classes. The values in parentheses reflect the percentage of these functional classes in the whole genome. Increase in percentage for metabolic pathways and lipid metabolism highlight the essentiality of these pathways. Proteins involved in metabolic pathways were further analysed using the scheme provided in Jamshidi *et al.*[45], to obtain a finer classification. Based on this (Figure 6b) four major classes emerged, amino acid biosynthesis pathways (30%), glycolysis (7%), purine metabolism (10%) and redox metabolism (8%). The importance of these pathways for maintaining active metabolism in a cell has been well understood through various biochemical studies [61-64]. Indeed proteins belonging to these pathways have been reported to be essential in other organisms as well [10,65,66].

**Figure 7:**
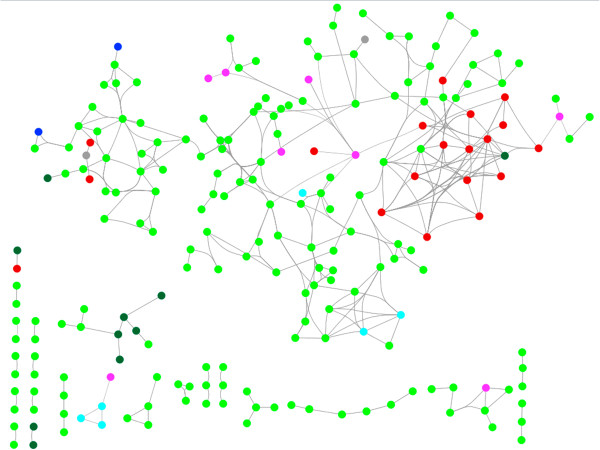
**A network view of the identified set of essential genes in *Mycobacterium tuberculosis*.** The set of 283 essential genes are mapped on the protein-protein interaction network to study the connectedness of the essential genes. The nodes are coloured based on the functional categories as provided in Tuberculist (blue: virulence, detoxification and adaptation; red: lipid metabolism; cyan: information pathways; dark green: cell wall and cell processes; light green: intermediary metabolism and respiration; pink: regulatory protein; grey: conserved hypotheticals). About 65% of the essential genes make a larger sub-network while the other nodes form smaller sub-networks.

To understand the functional roles of the 283 set of essential genes, they were viewed in context of the protein-protein interaction network used in this study (Figure 7). Of the 7844 edges present in the network, 641 edges are contributed by essential genes where both nodes contributing to an edge are essential as predicted by Equation 1. It is interesting to note that although these 641 edges form only 8.2% of all connections in the network, many of them form a connected sub-network by themselves. Each edge in the network indicates either a binding interaction or a functional linkage or both between a pair of proteins. The set of essential genes along with their inter-connections shown in Figure 7 can be regarded to form a core component of the cellular network, essential for that organism.

### Sequence and structural analysis

Biological function to any essential protein is imparted by its amino acid sequence and structure. Strong interdependence exists between protein essentiality, its function and the corresponding sequence and structural details of the protein. Therefore, an in-depth residue and structural level analysis is carried out for the final list of 283 proteins to obtain structural and sequence level insights that impart function and hence essentiality to the protein product of the gene.

### Residue level conservation

**Figure 8:**
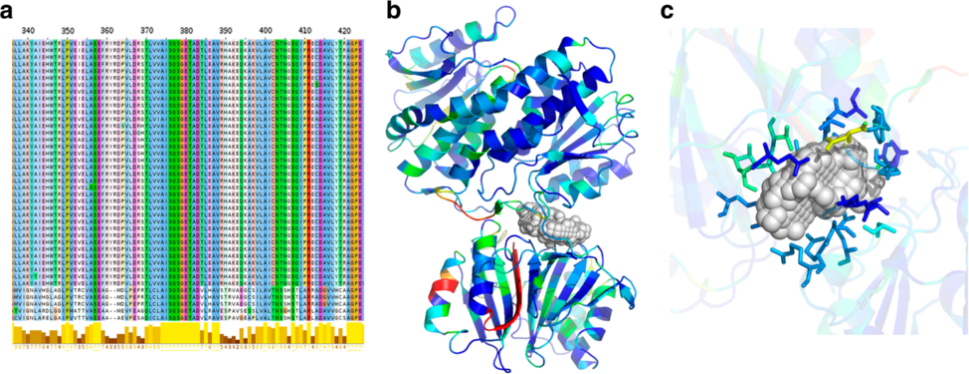
**Sequence and structural level details of Rv3436c (glmS). a)** Snapshot of MSA of this gene over the Mycobacterium genus is shown. Conservation scores for each residue in a protein are calculated using in-house algorithm; **b)** shows the protein structure of Rv3436c on which the predicted conserved residues are mapped. A colour scale (from blue to red denoting highest to the least) is used. **c)** One of the binding pockets of Rv3436c is shown and conserved residues that lie in the site are indicated. The pocket is shown in CPK representation and the residues forming the pockets are shown as stick model.

Certain residues in a protein are highly conserved across species. Such residues are conventionally taken to be functionally or structurally important for the protein [67]. Using multiple sequence alignments obtained from ClustalW [68], residue-wise conservation scores were calculated for each protein using a home-grown algorithm [69], as described in the methods section. This was carried out for each of the 283 proteins identified as essential. Sequence conservation analysis was obtained for 283 proteins, while structural analysis was obtained for 269 proteins, based on the availability of structural models. Figure 8 illustrates the nature of the analysis for these 269 proteins, by taking an example of Rv3436c (glmS), a glucosamine-fructose-6-phosphate aminotransferase. In each protein, the set of residues with a conservation score of ≥ 50% were considered for structural analysis.

**Figure 9:**
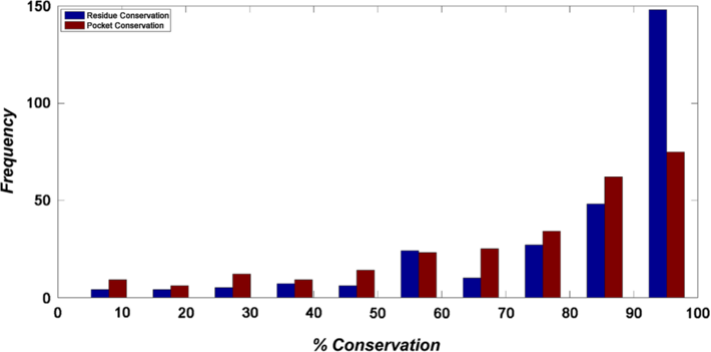
**Distribution of essential proteins based upon percentage of conserved residues seen in the protein sequence (blue bar) and those forming binding pockets (red bar).** X-axis represents the percentage of residues conserved (residues with conservation score ≥ 50, see Equation 8) in the protein (gene) or binding pockets and y-axis represents the frequency of these proteins in the final set studied (269 for structure analysis (red), 283 for sequence analysis (blue)). Essential genes are shown to be composed of high percentage of conserved residue and the corresponding pockets (red) are also shown to have more number of conserved residues.

Figure 9 (blue bar) shows the frequency distribution of the essential genes based on the percentage of residues conserved. As can be seen from the figure, more than 85% of the EGs have 70 – 100% residues conserved. Individual multiple sequence alignments in which conserved residues are highlighted are made available at http://proline.biochem.iisc.ernet.in/MtbEssentialGenes.

### Structural analysis

Binding sites in a protein structure are important for the function of the protein. Residues in a binding site are usually conserved and have functional importance attached to them. Using algorithms previously developed in the laboratory [70] binding sites were identified in the structural models for the set of EGs identified here. The models were obtained from *M.tb* structural proteome database, available in the laboratory [33]. Structural models were available for 269 of the 283 identified EGs, in which a total of 919 binding pockets were identified. The conserved residues identified from sequence conservation analysis were mapped on the binding sites or pockets. Figure 8c shows an example of binding pockets for Rv3436c and mapping of the conserved residues on it.

From this analysis, it was observed that all the 269 proteins had at least some conserved residues in their binding sites, amounting to 810 of the 919 sites studied. The binding sites were selected based on a consensus method that considered geometry, conservation as well as energy based cut-offs. This ensures that the identified pockets have sufficient volume and geometry to recognize a meaningful biological ligand. Figure 9 shows that more than 70% of the binding sites contain conserved residues. Considering the fact, that the set of sites studied includes crystallographically known binding sites as well as consensus site predictions, the extent of conservation is remarkable indeed. This was also verified statistically by calculating the quantile variation of conservation scores in the protein as a whole as compared to the binding sites alone in each case. The conservation of residues in the binding sites was significantly higher than for other regions in the protein (p-value < 2.2 e-^16^). The p-value was calculated between the conservation score distribution of the residues in the whole protein and that at the binding sites. Identification of these sites also provides a list of important residues and hence important interactions for the function of that protein. Our definition of essentiality implies that a given gene is essential if it has an essential function, which is conserved across other members in its close ecological niche. This means that the functional site residues that impart a function to a protein ought to be closely conserved. Hence the set of conserved residues in conserved pockets in the set of EGs indicate a basis for the conservation of function and hence for the essentiality of that protein.

## Discussion

The complexity of living organisms can be probed by understanding which genes play an essential role for the growth and survival and which are conditionally essential. Essentiality is studied here at multiple levels, at the levels of modules in the topological network of protein-protein interactions, at the pathway level by identifying essential metabolic, regulatory and signalling pathways, at the genome level, first by identifying high expression in multiple arrays of the same condition and second, by selecting genes that are evolutionarily conserved.

Consistent expression at a genome-scale level is used for weighting both the genome-scale metabolic network as well as the topological interactome, making the models resemble the actual experimental parameters more closely. An often asked question is whether gene expression levels can be used to understand protein abundance. While, gene expression may not always translate to corresponding protein abundances, there are many studies which show significant correlation between gene expression and protein abundances [71,72]. In any case, given the paucity of proteome-wide protein abundance data, this issue is difficult to address. In this study, we have tried to resolve this issue by considering multiple transcriptome profiles of essentially the same condition, simply to augment confidence levels. This is based on the premise that if a gene is consistently expressed across multiple profiles, it is unlikely to vary significantly in that condition and hence can be expected to correlate reasonably well with protein abundance. It should however be noted that focussing only on consistently expressed genes may not capture all genes that are essential for the organism, but in a systematic genome-scale study such as this, the decision has been to minimise false positives, with the belief that the subset of genes identified as essential should be of high confidence even if a few genes are missed out.

Two different types of systems approaches are used here, the interactome architecture analysis and the flux balance analysis of the genome-scale metabolic model, both integrating functional genomics data in terms of expression levels. The topological analysis utilizing graph theoretical methods of a weighted directed network explore the inherent organization of the connectedness of the individual molecular players in the cell and provide a basis to understand dynamics and the flow of chemical information using those connections. These approaches provide insights into the emergent properties arising in the system as a whole comprising specific interactions among the large number of components in a cell.

Four different computational approaches, each capturing a different piece of information are utilized in this study. The results from each are then compared to the three different experimental datasets available in literature. Table 1 provides the agreements between different pairs of methods and also among their combinations. The second and third column of Table 1 represent the coverage of each method. Comparison between individual methods is provided as a matrix in the same table (column 5-11). Rows 8-11 represents the comparison of individual methods with the three different Boolean logics used (Equations 1, 2, 3) as well as integrated approach. The values which are bold and italicized indicate the comparison between the experimental and theoretical methods. (MA = transcriptome, FBA = metabolic networks, PPI = protein-protein interaction network, PR = phyletic retention, IS = Integrated score, and experimental datasets are obtained from S = Sassetti *et al*. [18], G = Griffin *et al*. [16], Z = Zhang *et al.*[19]). Please note that conditional essentiality has not been mapped in this table.

**Table 1:** Detailed comparison of the various methods used to predict essentiality

**Sr. No.**	**Coverage**	**Shortlisted**		**MA**	**FBA**	**PPI**	**PR**	**S**	**G**	**Z**
*1*	*3923*	*2968*	**MA**	2968						
*2*	*663*	*319*	**FBA**	256	319					
*3*	*1240*	*323*	**PPI**	271	128	323				
*4*	*3923*	*1902*	**PR**	1490	173	164	1902			
*5*	*3923*	*654*	**S**	***544***	***160***	***143***	***340***	654		
*6*	*3923*	*774*	**G**	***654***	***200***	***169***	***427***	464	774	
*7*	*3923*	*688*	**Z**	***535***	***167***	***137***	***367***	390	530	688
*8*	*3923*	*283*	**Eq 1**	283	195	198	224	127	170	135
*9*	*3923*	*51*	**Eq 2**	51	51	51	51	31	38	30
*10*	*3923*	*139*	**Eq 3**	139	51	139	139	55	72	55
*11*	*3923*	*608*	**IS**	556	264	158	498	208	256	211

The table indicates comparison between different experimental and theoretical methods used for the study.

The three experimentally determined methods have identified 654 [18], 774 [16] and 688 [19] EGs. It must be noted that the set of EGs common to them are only 362, which corresponds to a commonality of only 47%, indicating lacunae with the experimental methods as well. Nevertheless for lack of any other comprehensive data, union of the EGs from these three methods, amounting to 1093 EGs are used for comparison with the predictions made in this study. The accuracies observed with individual methods are, 30.7% for phyletic retention analysis, 73% with FBA, 29.3% for gene expression and 62.2% with network analysis.

Various combinations of other Boolean operators were also implemented as shown in Additional file 10: Figure S5. When an intersection of positively identified proteins from all methods (Equation 2) was considered, only 51 EGs (Additional file 11: Table S6) could be identified as essential. Of these, 42 were found to be essential in the experimental datasets, while 3 of them were identified as essential under specific conditions, resulting in an accuracy of about 88.2%.

(2) EG=F∩P∩N∩M

Keeping in mind that the FBA model contained only a small set of genes (663), all belonging to metabolic class, an alternate Boolean logic was also implemented using only phylogenetic analysis, networks and microarray so as to remove the bias on metabolic genes caused by including FBA in the calculation and is described below,

(3) EG=P∩N∩M

139 genes were identified by this method and are listed in Additional file 11: Table S6. This set showed 60% correlation with experimental dataset. Overall, on comparing EGs identified by the different Boolean logics, Equation 1 described in the results, fares better as it a) has higher coverage as compared to Equation 2 and b) it is indeed not skewed towards any one method as seen from the analysis of Equation 3. Finally, the 283 EGs derived from Equation 1 were chosen as the final list from this study, which had an overall accuracy of 73.5%.

A detailed comparison of these Boolean logics with individual methods is also provided in Table 2. It is clear from the table that integrating different approaches into a meta-prediction yields better prediction capabilities than any of the individual methods. Thus the meta-prediction demonstrates the usefulness of combining systems architecture, metabolic dynamics and evolutionary insights along with gene expression. It is also interesting to note that some of the genes that are identified as essential from double knockout analysis using FBA, appear in the final list of EGs, as these were identified by phylogenetic and network analysis.

**Table 2:** Summary of datasets used in the study

**Dataset**	**Short description**
Boshoff *et al.*[37,81]	Transcriptome profiling of *M.tb* under different combinations of drug treatment using whole genome microarrays.
(GSE1642 from GEO)	
(transcriptome, FBA and network analysis)	
KEGG [51]	Database for linking genomic, proteomic and pathway level information. Also hosts resources for metabolites and ligands.
(network analysis)	
Reactome [85]	Open-source database for pathway information. It is manually curated and peer-reviewed.
(network analysis)	
STRING [84]	Provides a list of protein-protein interactions based on computational, high-throughput experiments and literature survey.
(network analysis)	
Wang *et al.*[86]	Protein – protein interactions were identified based on high-throughput bacterial two hybrid method.
(network analysis)	
Vashisht *et al.*[34]	*M.tb* interactome pathway re-annotated and reconstructed, containing a total of 1434 proteins linked through 2575 functional relationship.
(network analysis)	
Jamshidi *et al.*[45]	Reconstruction of metabolic network of the *in silico* strain of *M.tb*. Consists of 661 genes and 939 reactions. FBA was performed and essential genes were identified.
(FBA)	
Fang *et al.*[48]	Reconstruction of metabolic network of the *in silico* strain of *M.tb*. Consists of 663 genes and 1049 reactions. FBA was performed and essential genes were identified.
Anand *et al.*[33]	Structural models were obtained and validated for 70% of the *M.tb* genome. Fold based functional annotations were assigned and novel binding sites identified.
(binding site analysis)	
Sassetti *et al.*[18]	Transposon site hybridization was carried to identify set of essential genes.
(Validation)	
Griffin *et at.*[16]	High density mutagenesis and deep sequencing carried out to identify essential genes.
(Validation)	
Zhang *et al.*[19]	Identification of essential genes by dividing the whole genome sequence into windows of different length and performing TraSH analysis.

Further, as a way of removing reliance on any cut-off by any method, all 3923 genes in the genome were assigned a score based on an integrated scoring scheme (listed in Additional file 12: Table S5) devised to quantify the results obtained from different approaches to infer essentiality. 608 genes as obtained from the integrated score correspond to the top 50th percentile were also compared with individual experimental and theoretical method and is listed in Table 1. The highest confidence set of 51 EGs from Equation 2 were indeed the top 51 ranks based on the integrated score. Moreover, the final list of 283 genes identified by Boolean logic 1 (Equation 1) were also observed to have high ranks in this list, again demonstrating that there was no bias from any one approach.

Conditional essentiality has not been explicitly addressed in this study since the main focus was to identify those that are consistently essential in the WT condition. The same framework and techniques with small modifications can however be utilized, to probe condition-specific essentiality, when sufficient data becomes available. Yet, it is interesting to note that some of the EGs identified here are reported in literature to be conditionally essential genes based on individual experimental studies (indicated in Additional file 9: Table S7).

The final list of 283 genes was further compared to an earlier study by Raman *et al.*[73] that identified drug targets for *M.tb* based on an integrated pipeline approach. It is observed that about 31.4% of the essential genes identified in this study can also be used as drug targets for the pathogen. The subset of genes in *M.tb* belonging to fatty acid metabolism, purine metabolism, amino acid metabolism and redox metabolism are seen to dominate the list of essential genes.

Some of the genes belonging to the PE/PPE class are reported to be essential for *M.tb* H37Rv [16,17]. In this study, genes from this class were identified as essential from microarray analysis; however these were not identified by other methods. This is because, i ) FBA studies genes belonging to metabolic pathways only, ii) these class of genes are not evolutionarily conserved across the species and iii) no high confidence interaction data was available for these genes and thus was not included in the network. As a result, these genes were filtered out on applying the Boolean logic.

Extensive lists of essential genes identified using experimental whole-genome approaches are available in literature for several organisms such as *Staphylococcus aureus*[9,74], *Helicobacter pylori*[75], *Mycoplasma genitalium*[76], and *Pseudomonas aeruginosa*[77]. There are reports of such lists identified through computational approaches as well [8,78,79]. Such studies have primarily involved phylogenetic comparisons [1,9,22]. There are also a few reports recently, where flux balance analysis and related methods are used to study essential genes and reactions in metabolic networks [19,23,26,66]. However, apart from studies such as [27], there have not been sufficient efforts in the direction of integrated studies to infer essentiality. This study bridges perspective from several levels by considering the set of genes that are required for maintaining interactome integrity, maintaining metabolism and enabling growth as well as conservation through evolution of various organisms in the genus.

The set of genes identified have been probed further to predict amino acid residues that are important for the function of the gene product again by utilizing evolutionary information at a finer scale and integrating with the three dimensional structural information of protein molecules combined with the functional information of ligand binding pockets. The methodology used here can serve as a generic framework for identifying essential gene lists in other pathogens as well. The set of genes identified have the potential to be applied in drug discovery, taking target identification to a much more rational and wholistic level.

## Conclusion

A robust methodology to identify essential genes in *Mycobacterium tuberculosis* has been developed that integrates data obtained from different levels of abstraction. Towards this, microarray data has been used for essential gene prediction, knockout analysis using FBA to identify essential genes from metabolic networks, phylogenetic analysis to identify evolutionarily conserved genes and systematic knockout analysis of protein-protein interaction network has been performed. The results are validated using experimental datasets. Structural analysis of the proteins of the predicted essential genes is further analysed at the sequence and structural level to provide a basis for essentiality. Overall, the method recognises the importance of a multi–scale analysis and provides a framework for prediction of essential genes.

## Methods

Multiple approaches at different levels of abstraction are utilized in this study, as described in Figures 1 and 5. Methods for systems level analyses include flux balance analysis and topological analysis using graph theoretical methods of the protein-protein interaction network. Genomic microarray data from multiple sets has been analysed using microarray analysis. Sequence analyses and database searches are carried out using standard sequence alignment tools while structural analysis is carried out using algorithms previously developed in the laboratory. A summary of the datasets used in these analyses is given in Table 2.

### Microarray data analysis

An exhaustive transcriptome profiling for *M.tb* under many different conditions is publicly available in Gene Expression Omnibus (GEO) [80,81]. The GEO series, GSE1642 was selected for this study [37]. The authors have deposited the transcriptome profile of the pathogen under various drug treated and culture conditions. Of the 430 microarray sets available from this study, 39 samples that represented the control or WT conditions were hand-picked and the control chip (Cy3) was selected for the analysis. Since the focus here is to study gene essentiality under normal growth conditions, expression data from only the control samples were used. Cultures grown in MiddleBrook 7H9 media were considered as control and used for the analysis. Expression values for 3923 genes were available from this study.

A scoring scheme was formulated to score each gene in this dataset. Scores were defined so as to give high weightage to genes with high and consistent expression values. Thus the following equation was derived,

(4) $$S_{\mathrm{MA}\;}^{i}=\frac{\left(\sum_{j=1}^{39}x_{j}\right)}{A}\times 100$$

Where;

*i* = gene index

*j* = sample index

*x* = log2 normalised intensity value

$$A=\max_{i\in \mathrm{all}\;\mathrm{genes}}\left\{S_{\mathrm{MA}}^{i}\right\}$$

The shortlisted genes were further classified into functional classes using Tuberculist.

### Flux balance analysis

Genome-scale reconstructions of *M.tb* metabolism have been reported earlier [45,47]. The *i*NJ661 model was obtained from BiGG database [82], while the *i*NJ661v model was obtained from the study done by Fang *et al.*[48]. Fluxes through each reaction were calculated using the COBRA toolbox and glpk solver interfaced with MATLAB [83]. Single and double gene deletions were calculated using the COBRA toolbox. To make the computational model closer to the actual scenario, an integrated flux balance approach known as E-Flux was utilized [46]. In this, the median of gene expression values are used to bound the fluxes of each reaction in the model. Thus the relative ratios of the steady-state fluxes would indirectly take into account differences in protein abundances. The reaction fluxes, effects of single and double gene deletions were then computed using the COBRA toolbox.

### Network reconstruction

A weighted directed protein-protein interaction network was reconstructed consisting of 1240 nodes and 7844 edges. Data for protein-protein interaction was obtained from KEGG [51], STRING [84] and Reactome [85] databases and were manually curated to infer directions. The data used to build the network is provided in Additional file 6: Table S3. KEGG was mainly used to identify interactions among enzymes in the protein-protein networks. Any two proteins that shared a product-substrate relation were connected in the direction of the succeeding reaction and in cases of reversible reactions; edges were constructed in both directions. STRING uses a variety of computational and experimental methods to detect protein-protein interactions each of which is assigned a confidence score. To ensure minimum false positives, interactions with confidence score greater than 700 were included in the network. This criterion was relaxed for the experimentally verified interactions, where interactions greater than 500 were also included in the network. If the interacting partners formed a complex, bi-directional edges were constructed; else directions were defined based on inhibition/activation. Interactions were also obtained from a high-throughput experimental bacterial-2-hybrid study [86]. In order to minimise false-positive interactions, only a subset of this data that was reported by the authors to be experimentally verified by an independent method has been considered. Microarray data was integrated into the network using the median intensity value as the node weights in the network. The edge weights were calculated using the formula:

(5) $$E_{\mathrm{ij}}=\frac{1}{B_{\mathrm{ij}}\times \sqrt{N_{i}\times N_{j}}}$$

Where,

E_ij_ = edge weight between node i and j

B_ij_ = betweenness centrality of E_ij_

N_i_ = node weight of node i

N_j_ = node weight of node j

Visualization of the network and calculation of betweenness centrality for each edge was done using Cytoscape [87].

### Random network generation

To study the statistical significance of gene knockout analysis, random networks were generated using the ‘Random Network’ plugin in Cytoscape (apps.cytoscape.org/apps/randomnetworks). The networks were generated such that the degree distribution of the network was preserved. 100 such random networks were generated each with the same number of nodes (1240) as used in the original network. A second degree of randomness was added to the network, by weighing each node in the network with gene expression data randomly. For each random network, edge weights were calculated by randomising node weights and systematic knockout analysis performed to calculate the statistical significance (p-value) of gene knockout in the original network.

### Broken path analysis

Floyd–Warshall algorithm [88] was implemented in MATLAB using MatlabBGL toolbox to calculate all-pair shortest paths. All pair shortest paths were also calculated after systematic deletion of each node to study the effect of gene deletion on the network topology. Shortest paths for each perturbed network was compared to that of the WT to identify perturbations that led to the maximum disruption of the network. Disruption in the network was quantified based on the number of shortest paths through the given node that were completely broken (path length tends to infinity) or those where the path cost substantially increased. The total number of paths perturbed is calculated as follows:

(6) $$D_{i}=\frac{\sum PP_{i}}{\#\mathrm{path}s_{\mathrm{WT}}}\times 100$$

Where,

*D*_*i*_ = Disruption caused by KO of gene i

*PP*_*i*_ = Paths perturbed upon KO of gene i

*#paths*_*WT*_ = Total paths in the WT network

### Multiple sequence alignment

Protein sequences for all known mycobacterial species were obtained from the database UniProtKB (http://www.uniprot.org/help/uniprotkb), which resulted in a dataset of the genus *Mycobacterium* consisting of 63 different species and 195054 different proteins. Database searches using BLAST [52] was carried out for all proteins in *M.tb* H37Rv against the database of all mycobacterial species but by omitting all *M.tb* strains. Hits with identity ≥ 30%, sequence coverage ≥ 70% and e-values ≤ 0.001 were parsed for each query sequence. Multiple sequence alignments for each set were computed using ClustalW [68]. Phyletic retention scores were computed as follows:

(7) $$S_{P}^{i}=\frac{\mathrm{BLAST}\;\mathrm{hit}s_{i}}{N}\times 100$$

Where,

*BLAST hits*_*i*_ = number of unique species for which hits were obtained

*N =* number of species in the dataset = 63

### Residue conservation

These alignments were further used to obtain sequence level residue conservation scores. Calculation of a conservation score is similar to positional Shannon entropy evaluation, which is further normalized to scale from 0 to 100. Positional entropy is calculated as

(8) $$S\left(j\right)=\sum_{i\in \left\{\mathrm{AA}\right\}}-p_{i}\left(j\right)\times \log p_{i}\left(j\right)$$

Where,

*S*(*j*) = entropy calculated at sequence position j, where the sequence position is defined with respect to the multiple sequence alignment,

*i* = amino acids, in case of a gap insertion in the sequence alignment each amino acid is given equal count, while evaluating the frequency.

*p*_*i*_(*j*) = probability of occurrence of amino acid *i* in position *j*. This is calculated based on the MSA obtained

The above calculation is further normalized as provided in [89],

(9) $$s\left(j\right)=100\times \left(1-0.334\times S\left(j\right)\right)$$

### Structural analysis of ligand binding pockets

Crystal structures where available for the shortlisted proteins were obtained from PDB [90]. For others, high confidence structural models for the essential genes were obtained from the *M.tb* Structural Proteome database, where available [33]. The database provides putative binding sites or functional residues of *M.tb* protein structures, derived from a consensus approach using three different algorithms - (i) LIGSITEcsc [91], that utilizes solvent exposed surface conservation information, (ii) PocketDepth [70], an in-house geometric-based algorithm to detect cavities on protein surface and, (iii) SiteHound [92], a probe-based energy calculation method. A total of 269 structures were thus considered for this analysis.

All the pockets predicted by PocketDepth that were within the radius of 5 Å from LIGSITEcsc prediction and had an overlap with SiteHound predicted clusters were obtained. These pockets were further scanned to study the overlap between the residues forming pockets and the residues that are conserved as determined from MSA to derive the residue level attributes of an essential gene.

Integrated scores were also assigned to individual genes as follows:

Equation 4 was used to assign scores to each gene based on microarray data. For FBA, single gene deletions were performed and the grRatios obtained were further processed as described in Equation 10, to obtain scores for each gene.

(10) $$S_{\mathrm{FBA}}^{i}=\left(1-\mathrm{grRatio}\right)\times 100$$

Equation 7 was used as a score to rank genes based on phyletic retention and for network the following score was applied

(11) $$S_{N\;}^{i}=\frac{\left(\frac{PP_{\mathrm{KO}}}{\#\mathrm{path}s_{\mathrm{WT}}}\times 100\right)}{A}$$

Where,

*PP*_*KO*_ = number of paths perturbed upon knockout (inclusive of broken paths and paths with higher path cost)

*#paths*_*WT*_ = Total paths in the WT network$A=\max_{i\in \mathrm{nodes}}S_{N}^{i}$

Integrated scores was thus generated using the formalism given below

(12) $$IS_{i}=\sum_{j\in \left\{\mathrm{all}\;\mathrm{methods}\right\}}S_{j}^{i}$$

which is further normalized to obtain a percentile score.

## Competing interests

The authors declare that they have no competing interest.

## Authors’ contributions

NC conceptualised the idea and supervised the project. PB, SG performed microarray, FBA and network analysis while SM performed sequence analysis and PA carried out structural analysis. PB, SG and NC wrote the manuscript and all authors read and approved the final manuscript.

## Supplementary Material

Additional file 1: Table S1 List of genes shortlisted to identify essentiality from different methods. Click here for file

Additional file 2: Figure S1 Functional classification of the set of 2968 genes shortlisted by microarray analysis. Classification is based on Tuberculist annotations [39]. Different classes are indicated in the figure. Click here for file

Additional file 3: Figure S2 Comparison of single gene deletion study for *i*NJ661 (blue) and *i*NJ661v (red) models. X-axis represents only those genes that show an impact upon deletion. Y axis represents the impact of deletion (1 - grRatio). Value = 1 would mean no growth upon deletion and = 0 means no effect upon deletion. The impact for both the models are stacked on each other for a given gene index. It is noted that the effect of impact may differ between the models as seen by the length of the bar. Click here for file

Additional file 4: Figure S3 Pathway level classification of essential genes obtained from FBA analysis **a)** single gene deletion and **d)** double gene deletion. Click here for file

Additional file 5: Table S2 List of gene pairs identified as essential using double knockout gene deletion analysis in FBA. Different sheets contain genes obtained from the two models (sheet 2 and 3) and the union of both the models (sheet 1). Click here for file

Additional file 6: Table S3 Highly curated protein- protein interactions used to reconstruct the network. Click here for file

Additional file 7: Table S4 Detailed list of associations of all the nodes that forms the shortest path between Rv3441c and Rv1240 in WT (sheet 1) and KO (sheet 2) networks. Click here for file

Additional file 8: Figure S4 Functional classification of the set of 1902 genes shortlisted by phyletic retention analysis. Click here for file

Additional file 9: Table S7 List of genes identified as essential in this study and their comparison with those identified from experimental data reported in literature. *M.tb* proteins are identified by their Tuberculist accession numbers (for e.g. Rv0046c refers to gene-name, which is followed by agreement or lack of it, first from Sassetti *et al.* dataset (S) and then by Griffin *et al.* dataset (G) and finally with other datasets which identified condition dependent essentiality (C) (see text). A tick mark indicates that the gene is essential by the given experimental approach while a cross indicates that the gene is not essential by that method. S indicates that the gene is shown to cause reduction in growth rate (slow grower) [17]. * refers to genes that are shown to be essential under certain conditions. The table is colour coded based on the integrated scores assigned to each gene (see text). Shades of blue are used to classify genes with high, medium and low integrated scores, with dark blue representing genes that fall in the top 75^th^ percentile, and decreasing intensity of the shade represent 75^th^ to 50^th^ percentile and 50^th^ to 25^th^ percentile respectively. Click here for file

Additional file 10: Figure S5 Other Boolean logic equations applied to study essentiality; **a)** represents the most constrained and identified only 51 genes as essential; **b)** represents methods other than FBA and identifies only 139 genes. Figures are drawn using [93]. Click here for file

Additional file 11: Table S6 List of genes identified as essential using Equation 2 (sheet 1) and Equation 3 (sheet2). In the case of Equation 2, out of the 51 genes shortlisted, 42 correlated with experimental dataset while 3 showed conditional essentiality. The remaining 6 are written in bold and italicised. Click here for file

Additional file 12: Table S5 Integrated score for each gene is provided. The list also contains the scores obtained from individual methods. The last column indicates the correlation with experimental dataset. Click here for file
