# Supplementary figures and images for: A multi-level multi-scale approach to study essential genes in Mycobacterium tuberculosis
Source: BMC Syst Biol. 2013 Dec 5;7:132. doi: 10.1186/1752-0509-7-132 (PMC4234997; doi:10.1186/1752-0509-7-132)

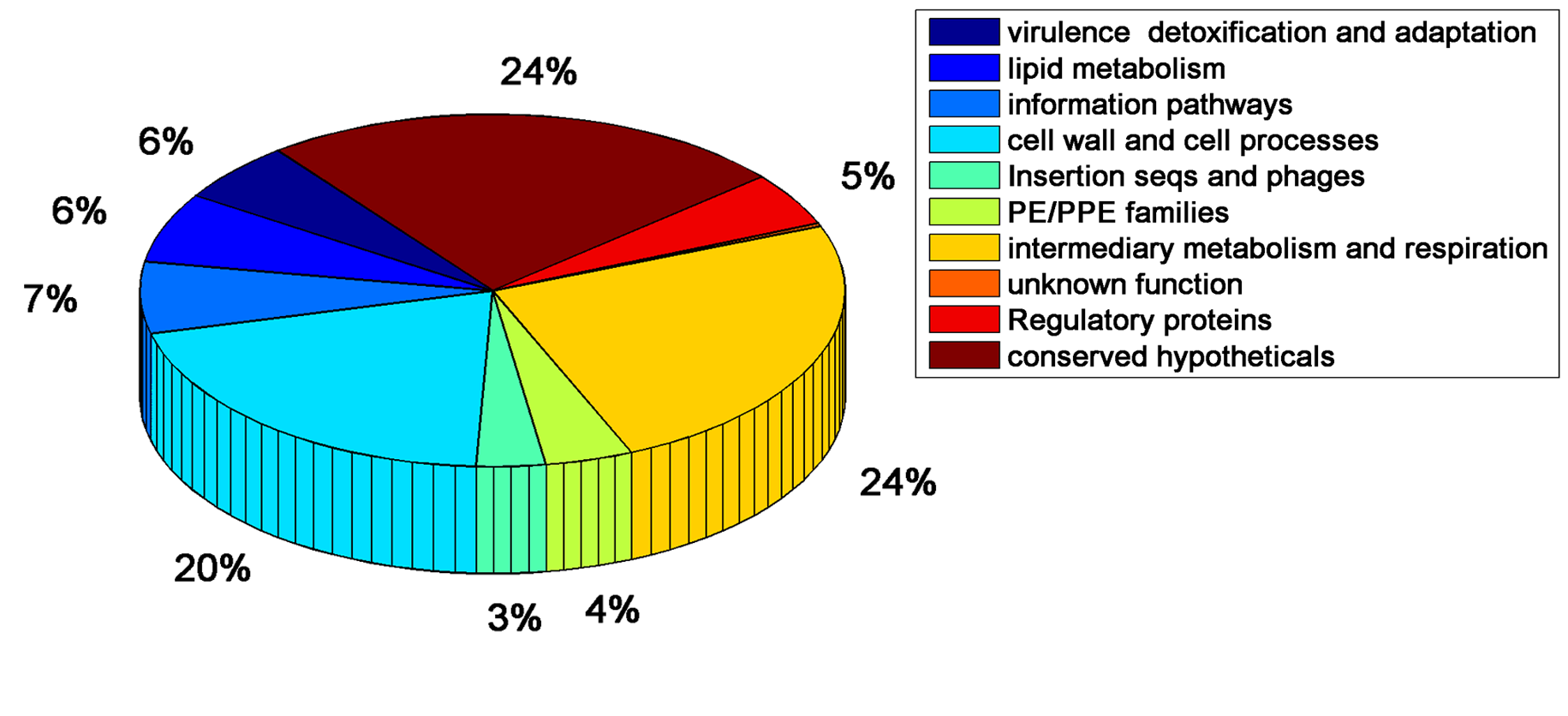

Supplement: Additional file 2: Figure S1 — Functional classification of the set of 2968 genes shortlisted by microarray analysis. Classification is based on Tuberculist annotations [39]. Different classes are indicated in the figure. [file 1752-0509-7-132-S2.tiff]

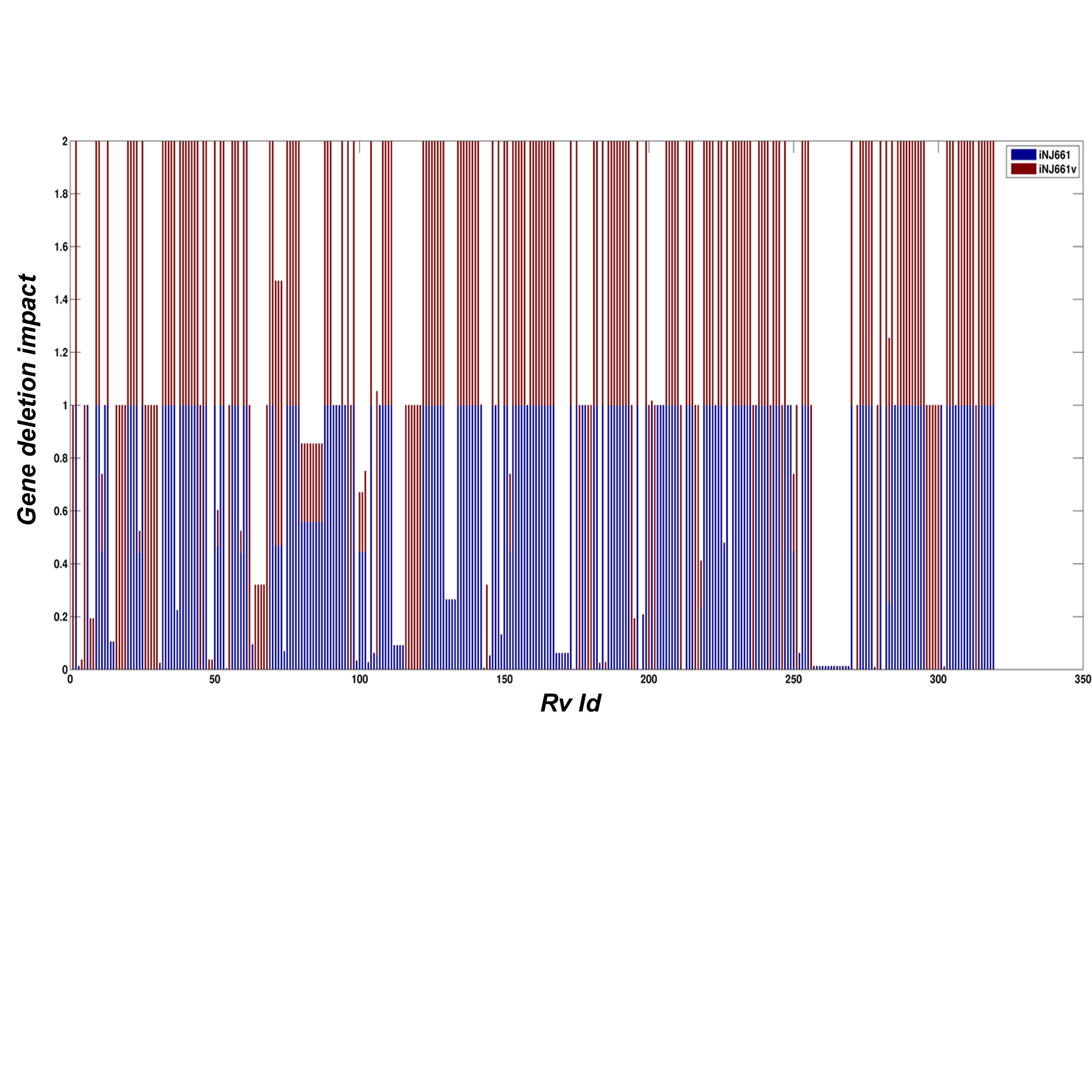

Supplement: Additional file 3: Figure S2 — Comparison of single gene deletion study for iNJ661 (blue) and iNJ661v (red) models. X-axis represents only those genes that show an impact upon deletion. Y axis represents the impact of deletion (1 - grRatio). Value = 1 would mean no growth upon deletion and = 0 means no effect upon deletion. The impact for both the models are stacked on each other for a given gene index. It is noted that the effect of impact may differ between the models as seen by the length of the bar. [file 1752-0509-7-132-S3.tiff]

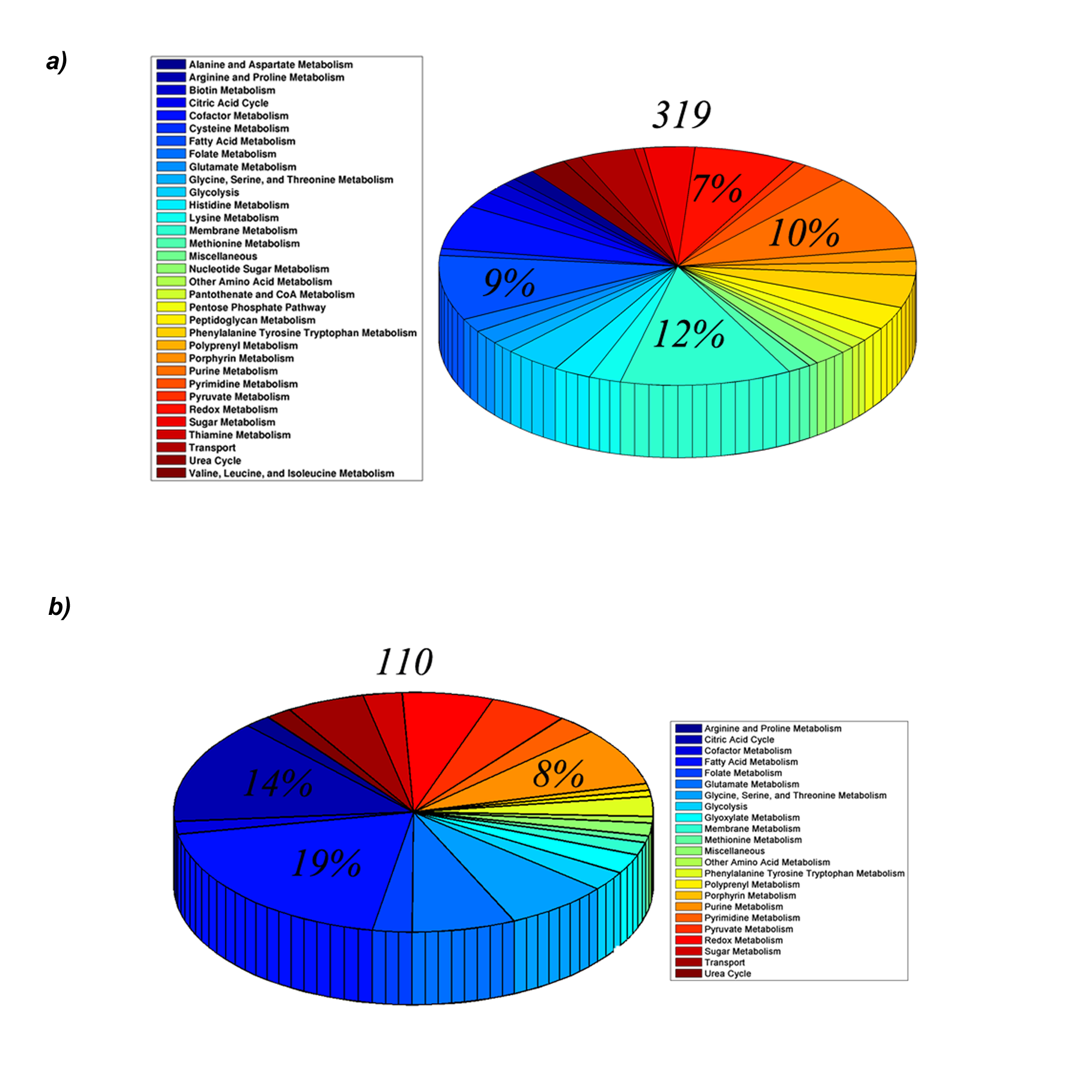

Supplement: Additional file 4: Figure S3 — Pathway level classification of essential genes obtained from FBA analysis a) single gene deletion and d) double gene deletion. [file 1752-0509-7-132-S4.tiff]

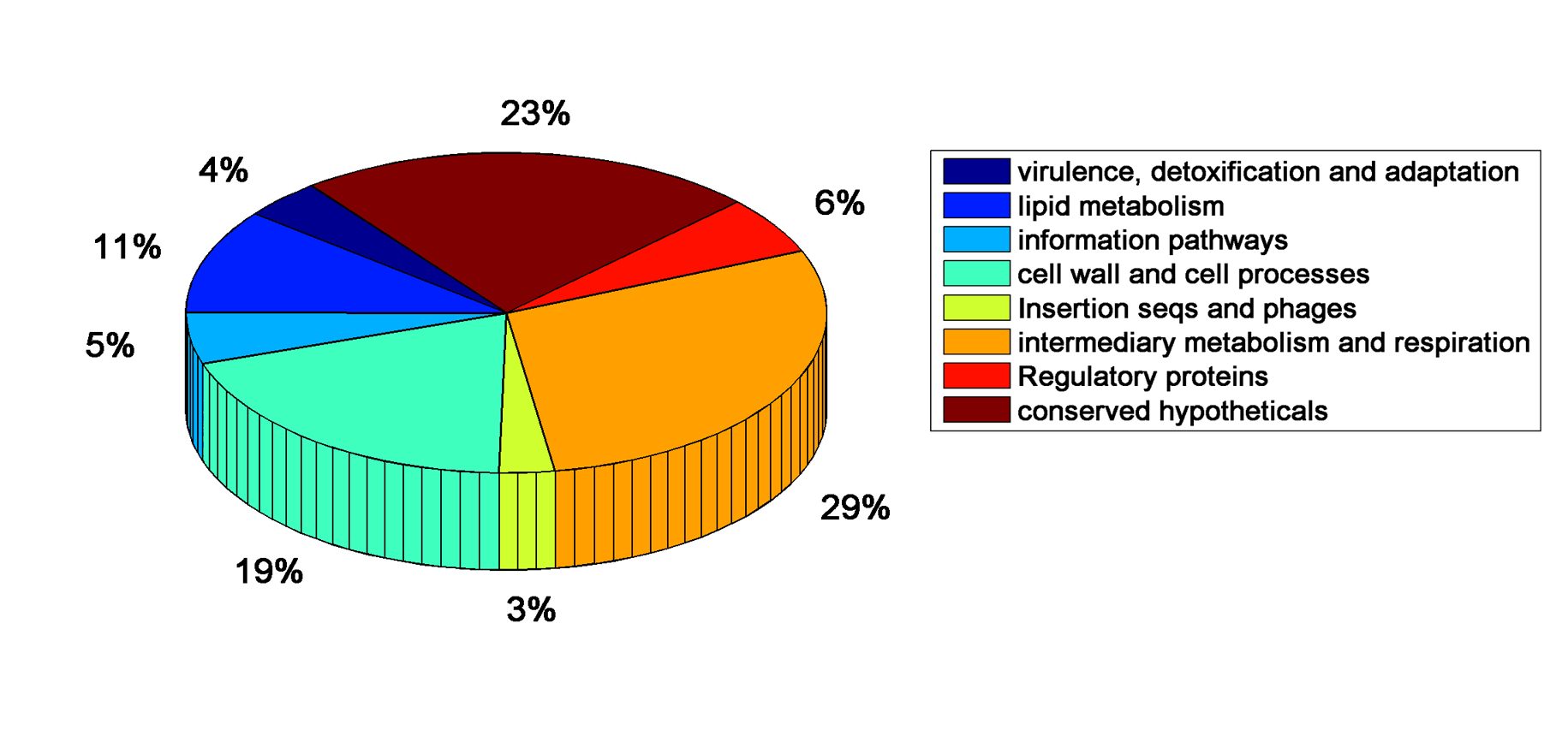

Supplement: Additional file 8: Figure S4 — Functional classification of the set of 1902 genes shortlisted by phyletic retention analysis. [file 1752-0509-7-132-S8.tiff]

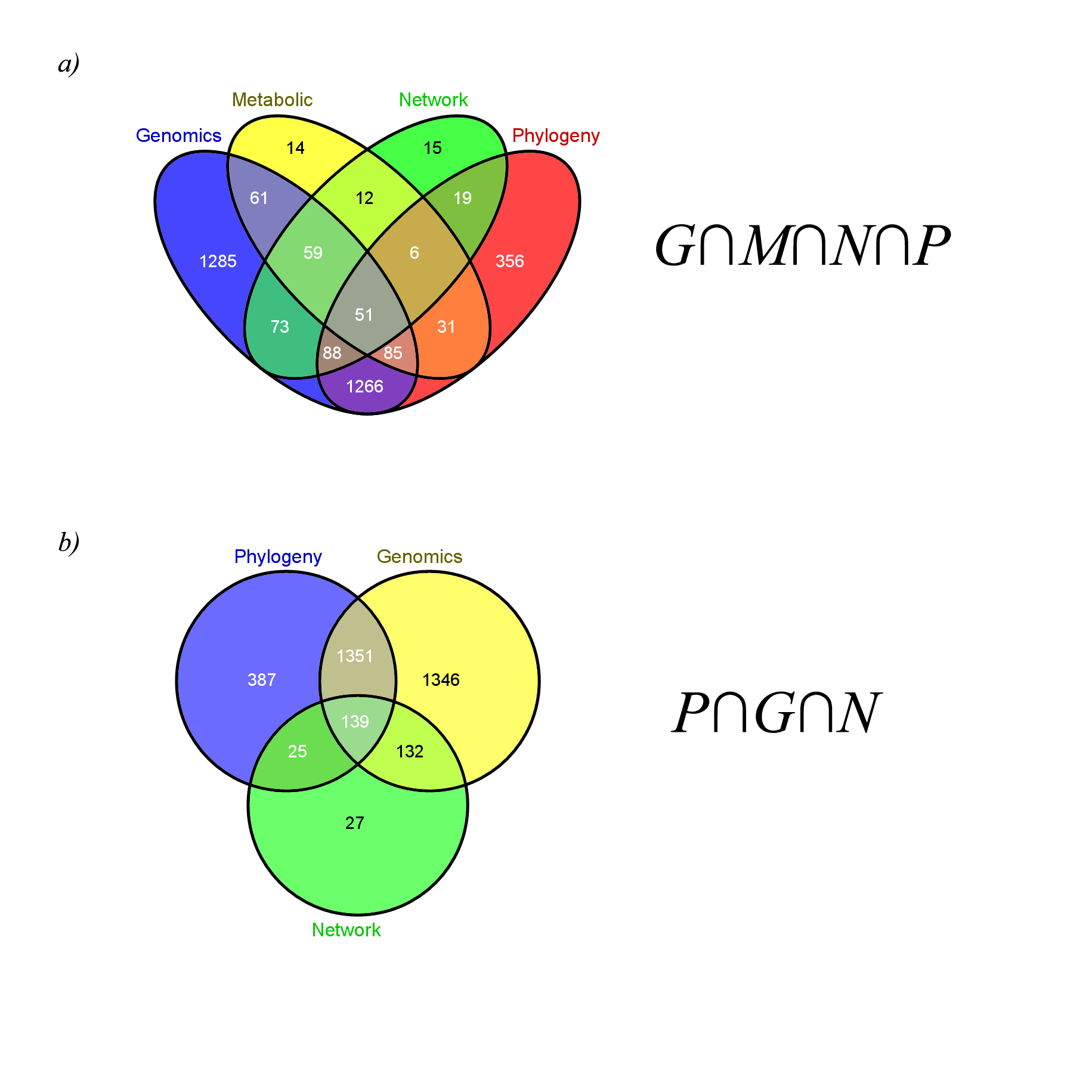

Supplement: Additional file 10: Figure S5 — Other Boolean logic equations applied to study essentiality; a) represents the most constrained and identified only 51 genes as essential; b) represents methods other than FBA and identifies only 139 genes. Figures are drawn using [93]. [file 1752-0509-7-132-S10.tiff]
